# Sustainable development goals: conceptualization, communication and achievement synergies in a complex network framework

**DOI:** 10.1007/s41109-022-00455-1

**Published:** 2022-03-14

**Authors:** Loredana Bellantuono, Alfonso Monaco, Nicola Amoroso, Vincenzo Aquaro, Angela Lombardi, Sabina Tangaro, Roberto Bellotti

**Affiliations:** 1 Dipartimento di Scienze Mediche di Base, Neuroscienze e Organi di Senso, Università degli studi di Bari Aldo Moro, 70126 Bari, Italy; 2 Istituto Nazionale di Fisica Nucleare, Sezione di Bari, 70125 Bari, Italy; 3 Dipartimento di Farmacia - Scienze del Farmaco, Università degli studi di Bari Aldo Moro, 70126 Bari, Italy; 4 Division for Public Institutions and Digital Government, United Nations Department of Economic and Social Affairs (DESA), New York, NY 10017 USA; 5 Dipartimento Interateneo di Fisica, Università degli studi di Bari Aldo Moro, 70126 Bari, Italy; 6 Dipartimento di Scienze del Suolo, della Pianta e degli Alimenti, Università degli studi di Bari Aldo Moro, 70126 Bari, Italy

**Keywords:** Complex networks, Sustainability, Sustainable development goals, Community detection, Natural language processing, Social networks, Multilayer networks

## Abstract

In this work we use a network-based approach to investigate the complex system of interactions among the 17 Sustainable Development Goals (SDGs), that constitute the structure of the United Nations 2030 Agenda for a sustainable future. We construct a three-layer multiplex, in which SDGs represent nodes, and their connections in each layer are determined by similarity definitions based on conceptualization, communication, and achievement, respectively. In each layer of the multiplex, we investigate the presence of nodes with high centrality, corresponding to strategic SDGs. We then compare the networks to establish whether and to which extent similar patterns emerge. Interestingly, we observe a significant relation between the SDG similarity patterns determined by their achievement and their communication and perception, revealed by social network data. The proposed framework represents an instrument to unveil new and nontrivial aspects of sustainability, laying the foundation of a decision support system to define and implement SDG achievement strategies.

## Introduction

In recent years, several emergencies on a global scale prompted policy makers and citizens to reconsider the established development model. Problems such as COVID-19 pandemic, global warming, the financial crisis, increasing inequalities in the access to medical assistance, food and education, highlighted the need for countries to undertake joint, fast and effective actions (Assembly 2020; Barbier and Burgess 2020). Since their establishment after World War II, specialized United Nations (UN) agencies play a role of coordination among member states in defining and implementing strategies to manage crises. An outstanding result of this kind of action is represented by the 2030 Agenda for Sustainable Development (Assembly 2015; Abud et al. 2017), a program endorsed by the 193 United Nations Member States (UNMS), based on five fundamental pillars: people, prosperity, planet, peace and justice, partnership. The 2030 Agenda is organized in 17 Sustainable Development Goals (SDGs) (Griggs et al. 2013), pursued through a series of actions and specific implementation policies, formalized into 169 targets (Guerrero and Castañeda Ramos 2020). The achievement level of each target, hence of the corresponding goal, is tracked and controlled in a transparent way, through a set of quantitative indicators (United Nations Statistics Division SDG API 2020). All countries are called to contribute to this collective effort towards sustainability, through the definition of their own development strategies. Specifically, the 2030 Agenda prescriptions should be translated into effective policies, projected according to the specific assets, the cultural background, the resources and criticalities of each country, which affect the urgency and the ease of achieving some goals instead of others. Due to such a variability in the starting conditions, diversified responses are registered in the SDG implementation at a country level (Sciarra et al. 2021; Assembly 2019, 2020, 2021; Biggeri et al. 2019; Sachs et al. 2020). Article 40 of the 2030 Agenda states that there should be no hierarchy of goals, and they should be globally pursued with the same priority level, up to the autonomy of each country in planning its own strategy towards sustainability.

The existence of different accomplishment patterns on a national scale sets a series of mutual similarities and differences among goals. In particular, we can say that a pair of goals is characterized by a strong *achievement similarity* if they show similar fulfilment levels for a large number of countries. A further similarity criterion between goals is related to their definition, based on the official statements of the respective targets: in particular, we can define a *conceptual similarity* between two goals, based on the semantic analogy between their formulations. Discrepancies between conceptual similarity and achievement similarity are particularly interesting, since they reveal the existence of several factors which affect the progress towards sustainability in nontrivial ways. In general, motivations behind the differences between the two kinds of similarity can be related to strengths and weaknesses of countries, political willingness, consciousness and sensitivity of public opinion towards a specific goal. The way in which a goal is communicated and perceived is actually crucial in determining the success of related initiatives, and social media represent an essential means for large-scale diffusion of information on goals, both by institutions and by the citizens. The latter are no longer passive recipients, but actively co-create communication on SDGs (Pilař et al. 2019; Malthouse et al. 2013), taking the chance of giving high resonance to their instances, and even condition the definition of strategies and policies for goal achievement. Based on these considerations, we introduce a third similarity criterion, that we call *communication similarity*, measuring the affinity in dissemination and perception of goals. Among all the possibilities in terms of media and type of data, we choose for practical reasons to focus on data available from Twitter: communication similarity between two goals is determined by the semantic analogy of the related tweets, identified through hashtags. Such a definition is similar to the one of conceptual similarity, but relies on an entirely different dataset. Though Twitter is not the most used social network in every country, it represents a particularly convenient and sound choice for two reasons: first, due to the tight length limit, tweets are typically characterized by a similar number of words (unlike, e.g., Facebook posts), which makes it reasonable to compare them to each other; second, use of Twitter is widespread, with all continents except Oceania represented in the list of twenty countries with the largest number of users (Statista Research Department 2021).

The above considerations qualify the set of the 17 SDGs as a complex system, in which multifaceted and nontrivial interactions among constituents occur. It is worth mentioning that such framework has already been employed in previous studies to inspect relations and spillover effects among goals (Guerrero and Castañeda Ramos 2020; Assembly 2020; Griggs et al. 2017; Pradhan et al. 2017; Fuso Nerini et al. 2019; Nilsson et al. 2018; van Soest et al. 2019; Sachs et al. 2019; Requejo-Castro et al. 2020; Tremblay et al. 2020). A tool of complexity science that allows to effectively analyse this kind of systems is provided by network models, which have been widely used in many real-world applications concerning a number of domains, such as economics (Pugliese et al. 2019; Hidalgo et al. 2007; Battiston et al. 2012; Amoroso et al. 2021), social sciences (Christakis and Fowler 2008; Bonaccorsi et al. 2019; Alessandretti et al. 2018), performance evaluation (Bellantuono et al. 2020), neuroscience (Sporns 2011; Amoroso et al. 2018, 2019; Bellantuono et al. 2021), genetics (Monaco et al. 2019, 2020), natural (Weathers and Strayer 2013; Cowen et al. 2006) and geological science (Poeppl et al. 2017), just to mention a few. Moreover, the complex network formalism has already been applied to the analysis of SDGs (Le Blanc 2015; Guerrero and Castañeda Ramos 2020), and, in particular, to the investigation of the interplay between goals and states that achieve them with their own specific strategies (Sciarra et al. 2021). In addition to these applicative outcomes, the theory of complex networks has experienced, in recent years, a remarkable development on the methodological level, which has led to the introduction of promising and innovative tools, such as multilayer networks (Bianconi 2018) and network potentials (Amoroso et al. 2020, 2021).

In this work we will investigate the ecosystem of interactions between the SDGs, comparing the structure of the similarity patterns related to three complementary aspects of the path towards sustainability: conceptualization and formulation, dissemination and perception, achievement and monitoring. In particular, we aim at detecting the presence of *central goals*, that are characterized, on average, by a pronounced similarity to the others, in terms of either conceptualization, communication or achievement. These goals play a strategic role, since they feature the maximal synergy with the rest of the SDG ecosystem, sharing a common ground even with goals that are weakly related to each other. In principle, centrality of a given SDG can either be a general property, or depend on the specific similarity criterion. In the latter case, it may happen that, e.g., a goal has a very sectorial official formulation, and therefore a low conceptual similarity with others, but instead exhibits a pronounced centrality in terms of communication or achievement.

**Fig. 1:**
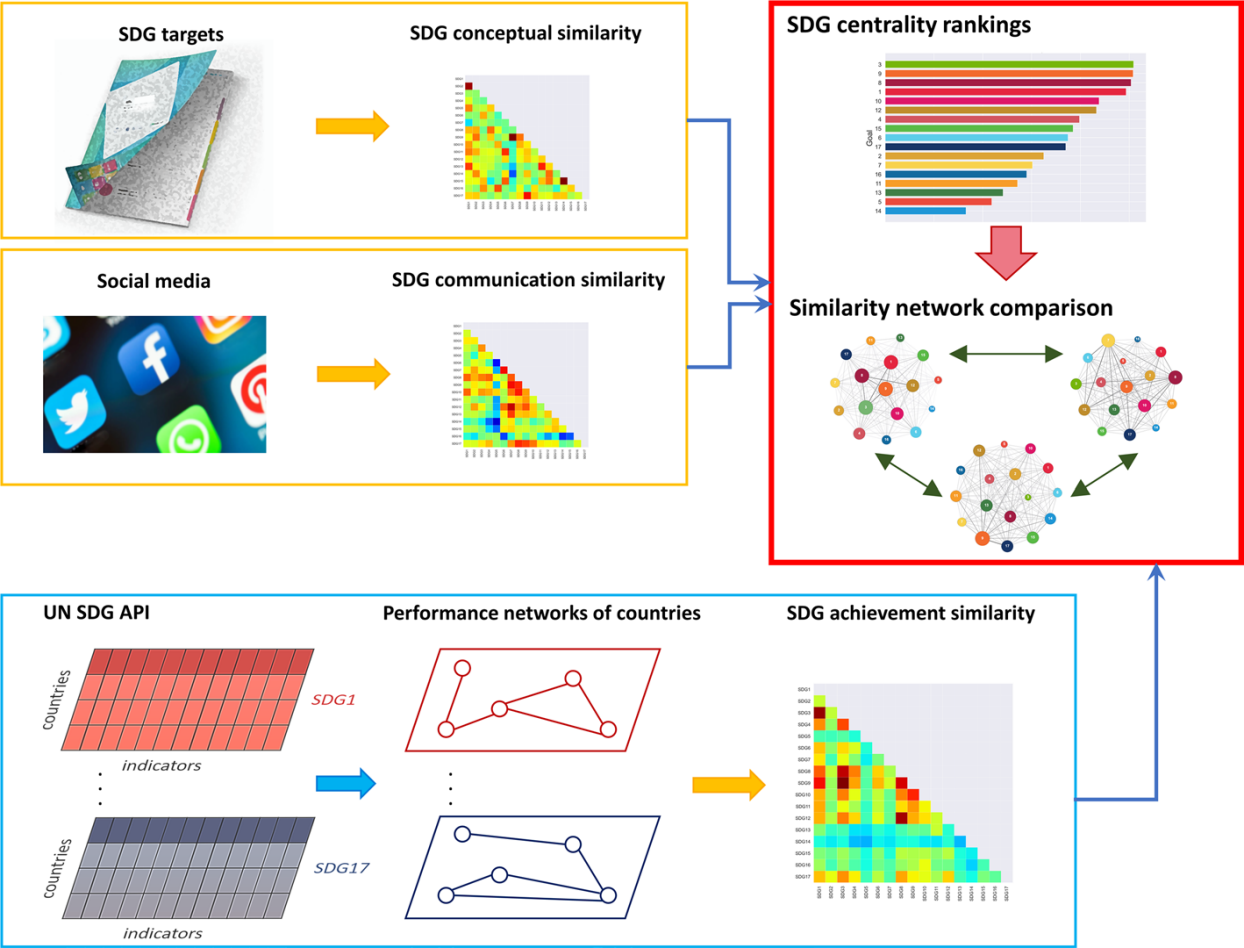
Schematic representation of the workflow followed in our research

The problem of finding central goals and characterizing the interplay among SDG similarities will be tackled by constructing a multiplex consisting of three layers, each containing 17 nodes representative of the goals, whose links are determined by the specific type of similarity. Conceptual and communication analogies between nodes will be determined through Natural Language Processing (NLP) techniques. Similarities in the achievement network, instead, will be established by constructing 17 *performance networks*, one for each SDG, in which nodes coincide with United Nations member states and their connections are determined by correlations in the related SDG achievement indicators. Comparison among these performance networks provides the basis to determine the achievement similarity between goals. A scheme of the described workflow is reported in Fig. 1. Though the investigation of inter-goal conceptual similarity has been tackled in previous works (see, e.g. (Fariña García et al. 2021; Lee and Jung 2019; Youn and Jung 2021)), our study is, to the best of our knowledge, the first one in which a comparison with achievement and communication similarity is performed. The insights emerging from our research, unveiling non-trivial synergies among SDGs, lay the foundations to develop a decision support system to define and implement sustainability-oriented policies.

## Results

We present the main properties of the three constructed networks, related to conceptualization, communication, and achievement of SDGs, and compare link weights and node centrality rankings in the three cases. Moreover, we use methods of multiplex analysis to characterize the role of each node across the layers.

**Fig. 2:**
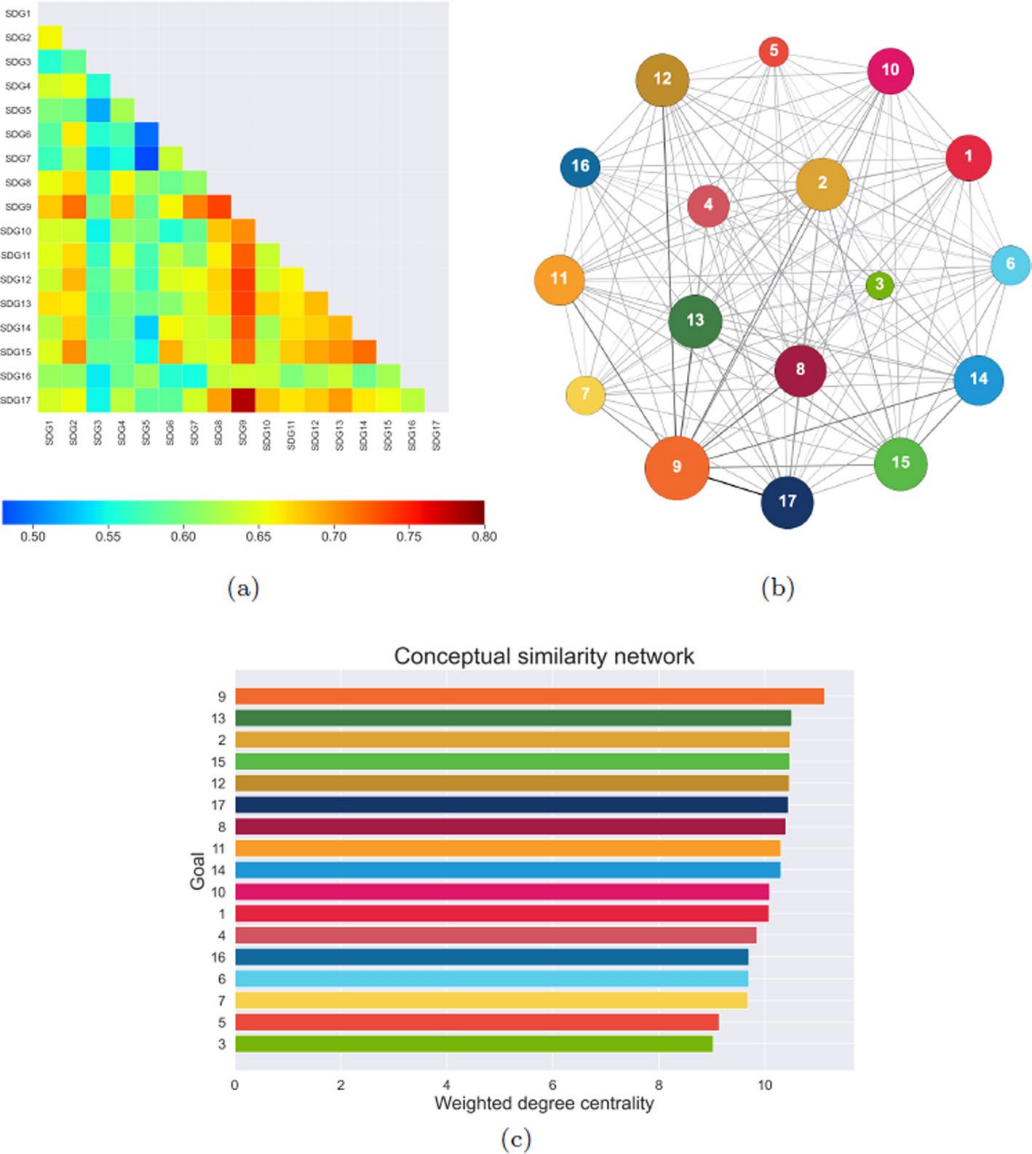
**a** Heatmap representing the weight of links between each pair of goals in the conceptual network. **b** Graph representation of the conceptual network, with node size related to their weighted degree centrality and edge thickness proportional to their weight. **c** Bar plot of the weighted degree centrality of nodes in the conceptual network; values are reported in the “Appendix”

### Conceptual network: inter-goal similarities from target semantic content

We investigate the conceptual analogies among SDGs by means of a complex network that reproduces semantic similarities between their targets. This network, complete by construction, consists of 17 nodes connected to each other by links whose weights are determined by a conceptual similarity measure; details on the computation of such quantity are reported in Materials and Methods (*Semantic similarities among text items* subsection). The similarity values, represented in the heatmap of Fig. 2a, range between 0.48 and 0.78. Considering that the measure is defined in the interval $[-1,1]$, this result indicates an average analogy between target statements. The strongest connections involve Goal 9 (Industry, innovation and infrastructure) with, in decreasing order of weight magnitude, Goals 17 (Partnerships for the goals), 12 (Responsible consumption and production), 13 (Climate action), 8 (Decent work and economic growth) and 14 (Life below water). On the other hand, the weakest similarity relations are established between goals concerning very diverse sectors of sustainable development, whose semantic areas show a limited overlap. In particular, Goal 5 (Gender equality) is connected to Goal 7 (Affordable and clean energy) and Goal 6 (Clean water and sanitation) with the lowest and second-lowest link weight, respectively. The network structure is reported in Fig. 2b, with node size related to their weighted degree centrality, and edge thickness proportional to their weight. The apparent semantic centrality of Goal 9 is formally confirmed by the ranking of SDGs weighted degree centrality, reported in Fig. 2c, computed as the sum of link weights connected to each node. The second and third most connected nodes represent Goal 13 and Goal 2 (Zero hunger), respectively, while nodes associated to Goal 7, Goal 5 and Goal 3 (Good health and well-being) are characterized by the lowest values of centrality.

**Fig. 3:**
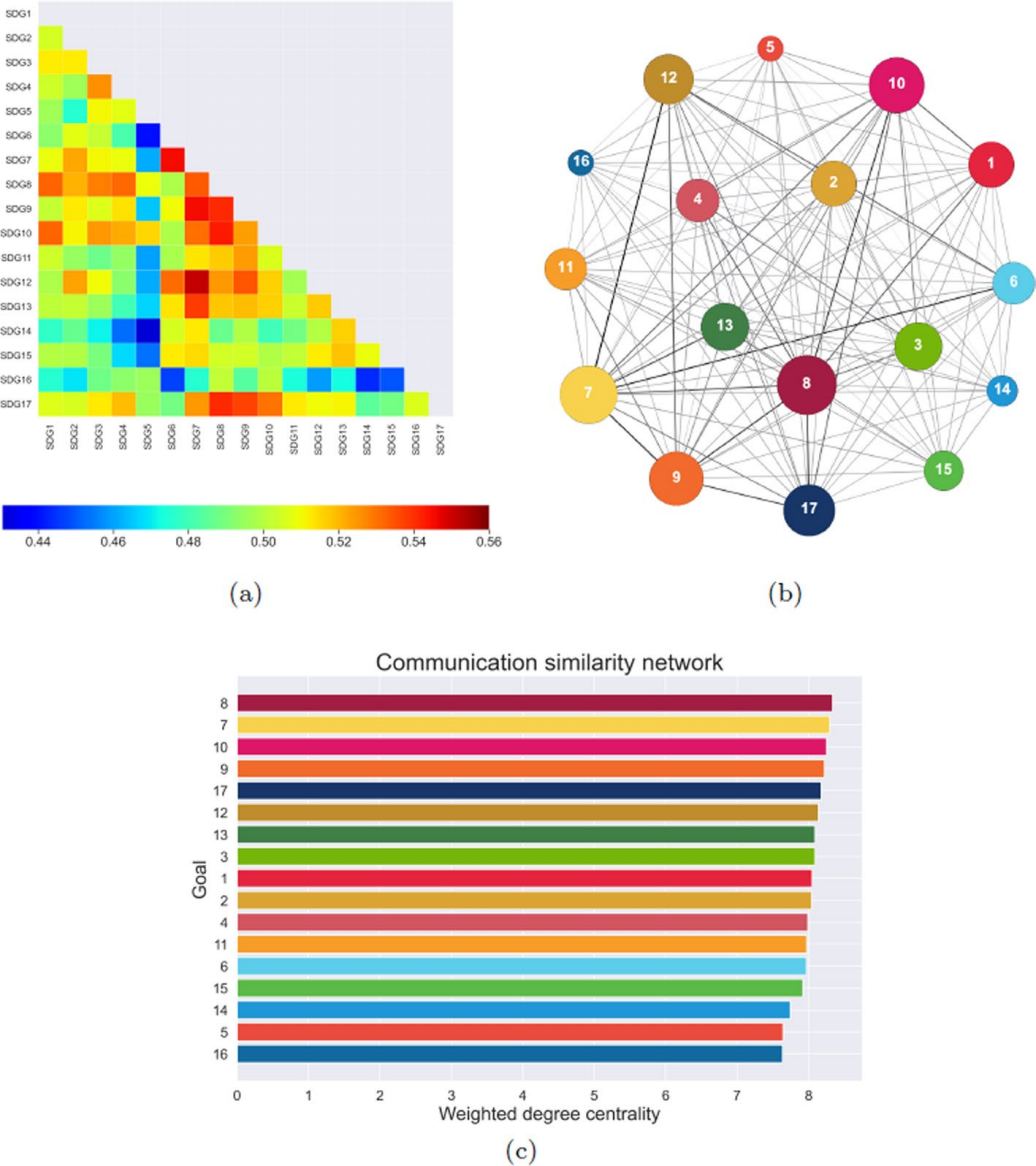
**a** Heatmap representing the weight of links between each pair of goals in the communication network. **b** Graph representation of the communication network, with node size related to their weighted degree centrality and edge thickness proportional to their weight. **c** Bar plot of the weighted degree centrality of nodes in the communication network; values are reported in the “Appendix”

### Communication network: inter-goal similarities from Twitter data

The *communication network* is based on text similarity as well. However, in this case the source of comparison between goals is not contained in official documents, but in posts on the popular social network Twitter. We consider both dissemination tweets by official accounts and communication tweets by common users, containing hashtags related to one or more specific goals (details on tweet selection in the *SDG tweets* subsection of Materials and Methods). The 17 nodes are connected according to the semantic similarity between tweets that mention the corresponding SDGs. The similarity values of the resulting network, reported in the heatmap of Fig. 3a, range between 0.43 and 0.55, showing an homogeneity of connections even higher than the one observed in the conceptual network. The link of largest weight connects Goal 7 (Affordable and clean energy) and Goal 12 (Responsible consumption and production), that are sometimes even mentioned in the same tweet. Goal 7 has a strong similarity also to Goal 6 (Clean water and sanitation) and Goal 9 (Industry, innovation and infrastructure). Other high weights are involved in the connection of Goal 8 (Decent work and economic growth) with Goal 10 (Reduced inequalities) and Goal 17 (Partnership for the goals). The least connected pair involves Goal 5 (Gender equality) and Goal 6, intuitively unrelated to each other. Among the weakest links we find those connecting Goal 5 and Goal 14 (Life below water), Goal 14 and Goal 16 (Peace, justice and strong institution), Goal 6 and Goal 16, Goal 15 (Life on land) and Goal 16. The network structure, with node size related to their weighted degree centrality and edge thickness proportional to their weight, is reported in Fig. 3b. The recurrence of Goal 16 among the weak connections is confirmed by the fact that, as shown in Fig. 3c, it ranks last in weighted degree centrality. The most central goals are, instead, Goals 8, 7 and 10, all appearing as one of the nodes in the top-weight network connections.

**Fig. 4:**
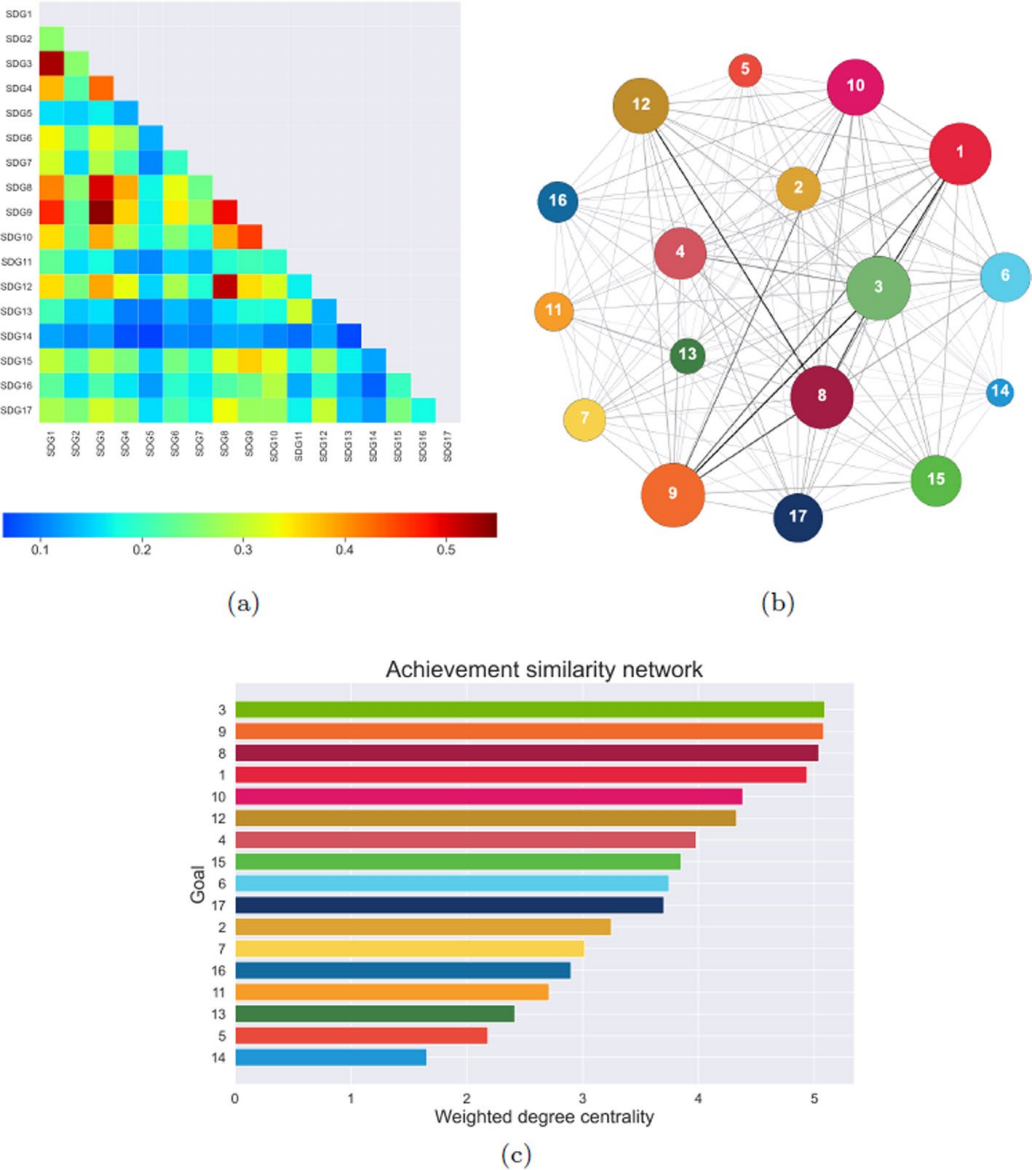
**a** Heatmap representing the weight of links between each pair of goals in the achievement network. **b** Graph representation of the achievement network, with node size related to their weighted degree centrality and edge thickness proportional to their weight. **c** Bar plot of the weighted degree centrality of nodes in the achievement network; values are reported in the “Appendix”

### Achievement network: inter-goal similarities from country achievements towards sustainability

The *achievement network*, whose nodes represent the 17 SDGs, is constructed by comparing the performances reached by different countries in each pair of goals. The procedure to determine such network, described in greater detail in Materials and Methods, requires as a preliminary step the construction of 17 *performance networks*, one for each goal, in which nodes coincide with UN member states, while weighted links quantify similarity across countries in the indicators related to the specific goal. The performance networks are then compared to each other through the Weighted Jaccard (WJAC) similarity, whose values provide the weights of links in the achievement network. The heatmap in Fig. 4a shows the magnitude of such weights for each pair of goals. The minimum similarity 0.063 is recorded between Goals 5 (Gender equality) and 14 (Life below water), which are intuitively uncorrelated and both characterized by generally weak connections. The maximum achievement similarity 0.542 is reached by Goal 3 (Good health and well-being) and Goal 9 (Industry, innovation and infrastructure), which are also characterized by high link weights with other goals. Among the strongest connections are, in descending order of weight magnitude, those between Goal 3 and Goal 1 (No poverty), Goal 8 (Decent work and economic growth) and Goal 12 (Responsible consumption and production), Goal 3 and Goal 8, Goal 8 and Goal 9. The network structure, with node size related to their weighted degree centrality and edge thickness proportional to their weight, is reported in Fig. 4b. As in the case of the conceptual similarity network, Goal 9 is one of the most central, being second in the weighted degree centrality ranking reported in Fig. 4c. Other central SDGs are 3, 8 and 1. On the other hand, Goal 13 (Climate action) has one of the lowest centrality values, which is in contrast with its pivotal role in the conceptual similarity network. Goal 5, instead, exhibits a peripheral nature, namely a low centrality value, in all networks. The result of Goal 14, which ranks last, is consistent with the low centrality observed in the communication network.

### Comparing inter-goal similarity patterns

We compare the similarity patterns emerging from the three networks, with a special focus on the centrality hierarchies of their nodes. First, as reported in Table 1, we compute the Spearman (Zwillinger and Kokoska 2000), Pearson (Kendall 1961), and Kendall (Kendall 1938) correlations between the link weights pertaining to each pair of networks. The results show that significant correlations exist between the two networks based on NLP, and between the communication and the achievement network. On the other hand, correlation between link weights in the conceptual and the achievement network is not statistically significant. We also compare the sets of weighted degree centrality values related to each SDG network (see full list in “Appendix”) by computing their mutual Spearman, Pearson, and Kendall correlations. The result of this analysis, reported in Table 2, is unanimous. The conceptual and the achievement networks are very weakly correlated to each other. The pair formed by conceptual and communication network is characterized by mutual correlations that are higher but not statistically significant, as their *p*-values do not allow us to reject the null hypothesis of no correlation. Interestingly, only the weighted degree centrality values in the communication and achievement networks are correlated in a statistically significant way, with $p<0.01$ for the Pearson correlation and $p<0.05$ for the Spearman and Kendall ones. To further investigate the role of nodes in the three networks, corroborating the results on centrality hierarchies, we also compute the multiplex participation coefficient. This metrics, defined and characterized in Materials and Methods, quantifies the tendency of a node to have similar relevance in all the layers of the multiplex; detailed results for the 17 SDGs are reported in the “Appendix”.

**Table 1:** Different types of correlation (Spearman, Pearson, Kendall) between link weights in the three pairs of networks, with the related *p*-values

	Conceptual-communication	Conceptual-achievement	Communication-achievement
Spearman	$\mathbf {0.450}$ ($p = 4\cdot 10^{-8}$)	0.109 ($p = 0.206$)	$\mathbf {0.450}$ ($p = 4\cdot 10^{-8}$)
Pearson	$\mathbf {0.495}$ ($p = 9\cdot 10^{-10}$)	0.103 ($p = 0.232$)	$\mathbf {0.456}$ ($p = 3\cdot 10^{-8}$)
Kendall	$\mathbf {0.316}$ ($p = 5\cdot 10^{-8}$)	0.076 ($p = 0.192$)	$\mathbf {0.310}$ ($p = 9\cdot 10^{-8}$)

Statistically significant correlations are highlighted in boldface

**Table 2:** Different types of correlation (Spearman, Pearson, Kendall) between weighted degree centrality values in the three pairs of networks, with the related *p*-values

	Conceptual-communication	Conceptual-achievement	Communication-achievement
Spearman	0.272 ($p = 0.291$)	0.054 ($p = 0.837$)	$\mathbf {0.549}$ ($p = 0.022$)
Pearson	0.381 ($p = 0.131$)	0.134 ($p = 0.607$)	$\mathbf {0.625}$ ($p = 0.007$)
Kendall	0.191 ($p = 0.308$)	0.044 ($p = 0.839$)	$\mathbf {0.382}$ ($p = 0.034$)

Statistically significant correlations are highlighted in boldface

## Discussion

The results of the proposed analysis unveil the different relevance of SDG synergies in terms of conceptualization, communication and achievement. Large values of weighted degree centrality in the conceptual layer highlight similarities that are implicitly incorporated in the official target formulations, thus reflecting the initial expectations of policy-makers on inter-goal connections. Centrality in the communication layer, instead, provides relevant information for SDG dissemination. Actually, such centrality points out goals that, being frequently associated with others by social network users, can serve as drivers to promote other multiple objectives. Finally, centrality in the achievement layer unveils those SDGs whose implementation is particularly compatible with the other ones, and can thus be strategic in designing coordinate actions to pursue more tasks together. On the other hand, weakly connected nodes in the achievement network are in no way less relevant than the most central ones, but require dedicated effort. Considerations on SDG centralities within each layer are integrated with information on the multiplex participation coefficient.

Goal 9 (Industry, innovation and infrastructure) has a pivotal role in the conceptual network, which indicates that the themes contained in the related targets have a frequent semantic matching to the other goals’ topics, that implicitly refer to innovation as a driver of development. The relevance of Goal 9 is utterly confirmed by the results for the other layers, since it ranks among the nodes with highest centrality in both of them. In particular, its synergy with different aspects of development is confirmed by the result in the achievement network ranking, in which Goal 9 is very close to the top. The other SDGs that reach the top half of all the centrality rankings are Goal 8 (Decent work and economic growth), particularly relevant in the communication network, and Goal 12 (Life on land). All the aforementioned nodes rank among those with the highest multiplex participation coefficient, which confirms that each of them plays equally important roles in the conceptual, communicative and achievement networks.

Goal 3 (Good health and well-being) and Goal 1 (No poverty) are much more relevant in the achievement network than in the other ones, in particular the conceptual network. This result, corroborated by the relatively lower values of multiplex participation, shows that, though these SDGs are not strongly semantically correlated to other goals, they have important synergies with development achievements in different sectors. This result is not surprising, considering the essential role of health and absence of poverty as proxies of general development.

The centrality of Goal 13 (Climate action) and Goal 2 (Zero hunger) in the conceptual network has no strong counterpart in the other layers. Goal 7 (Affordable and clean energy) represents another outstanding case, since it is frequently associated to other goals by social network users, but much less connected in terms of conceptualization and achievement. A possible explanation for the oversized communication centrality of Goal 7 can be related to the bias due to the more widespread use of social networks in developed countries, where users are more sensitive to the matter of clean energy and tend to associate it to other sustainable development topics.

Finally, it is worth mentioning two SDGs that exhibit weak connections in general, namely Goal 5 (Gender equality) and Goal 16 (Peace, justice and strong institutions), which are much more focused on civil rights and politics compared to the other goals. These goals also rank among those with the lowest multiplex participation coefficient, since they have comparatively negligible connections in one (for Goal 16) or two layers (for Goal 5). Being a sectorial goal, however, does not imply poor ability to raise engagement, as testified by the fact that Goal 5 ranks second in terms of collected tweets in the reference period (see Materials and methods), after another goal with a social focus such as 4 (Quality education).

The comparison of SDG centrality rankings, in terms of Pearson, Spearman and Kendall correlations, unanimously shows that the one relating communication and achievement networks is the only statistically significant correlation. This result is particularly striking, since it shows that two networks constructed with completely different procedures are more similar to each other than the two networks based on NLP. Remarkably, statistically significant similarity of centrality rankings exists between the networks built from two *a posteriori* phenomena, though very different from each other: the spontaneous expression of users on a social network, and the results achieved by countries. On the other hand, no statistically relevant correlation is found with node centralities in the conceptual network, that is constructed *a priori*, namely using official statements that were issued before the quest for SDGs started.

We remark that the results on network centrality are not meant to highlight more or less hidden hierarchies of importance among SDGs, which would also go against the official statements. Instead, our study points out the transversality, on different grounds, of specific goals, which constitutes a relevant asset in view of accomplishing the SDG roadmap. Actually, a central node in conceptualization, communication, or achievement, can have a driving effect on the other ones. Since centrality rankings depend on the specific criteria of network construction in each layer, it would be desirable to exploit central goals in different domains, with their own peculiarities and pivotal connections, to promote the realization of the 2030 Agenda.

## Materials and methods

### Data collection

We report here the sources of data employed in the construction of the three SDG similarity networks, and describe preliminary processing steps.

#### SDG targets

The 17 Sustainable Development Goals are articulated in a series of 169 targets (Sustainable Development 2021), aimed at identifying well-defined objectives and tasks for each goal. Targets provide the basis to define indicators, that allow to quantify progress of countries towards achievement. Table 3 reports the number of targets pertaining to each SDG.

**Table 3:** Data available for each goal

Goal	Title	Targets	Tweets	Indicators
1	No poverty	7	1809	75
2	Zero hunger	8	3928	36
3	Good health and well-being	13	5383	126
4	Quality education	10	10484	404
5	Gender equality	9	9556	156
6	Clear water and sanitation	8	5433	34
7	Affordable and clean energy	5	9976	10
8	Decent work and economic growth	12	2631	158
9	Industry, innovation and infrastructure	8	1472	24
10	Reduced inequalities	10	1710	65
11	Sustainable cities and communities	10	3333	28
12	Responsible consumption and production	11	1884	69
13	Climate action	5	3524	13
14	Life below water	10	3702	8
15	Life on land	12	1611	23
16	Peace, justice and strong institutions	12	6256	65
17	Partnerships for the goals	19	2378	52

Number of targets, tweets and performance indicators collected for each goal, to construct the conceptual, communication and achievement networks

#### SDG tweets

We perform tweet scraping using the *snscrape* (JustAnotherArchivist 2020) and *tweepy* (Roesslein 2020) Python libraries, with the latter providing the access keys to the API used for scraping. We construct a query to retrieve each tweet satisfying the following requirements:

- containing a text string of the type “SDG *n*”, “SDG*n*”, “#SDG *n*”, or “#SDG*n*”, with *n* a number between 1 and 17;
- having been posted by a verified user;
- having been published between 30 June 2011 and 30 June 2021;
- having been written in English.

The scraping operation provides 89,270 tweets, then subjected to the filtering process described in subsections *Preprocessing text data* and *Removing spurious tweets through semantic similarity with SDG titles*. Notice that tweets are collected as many times as a different SDG is mentioned therein; therefore, a tweet can be counted more than once if it contains reference to various SDGs.

#### SDG performance indicators

SDG performance indicators are used to construct the 17 performance networks of countries. Their values, updated at 3 November 2020, have been retrieved from the SDG API [7]. We report in Table 3 the number of indicators considered for each goal.

### Semantic networks of SDGs

We construct two kinds of semantic networks; in both of them, nodes represent goals and weighted links are related to the semantic similarity between them. The two networks are respectively based on conceptual similarity, determined by measuring the semantic analogy between targets, and communication similarity, determined by considering affinity of tweet contents.

#### Preprocessing text data

We analyze textual data related to targets and tweets with Natural Language Processing techniques, employing the *nltk* (Bird et al. 2009) and *gensim* (Řehůřek and Sojka 2010) libraries. In particular, text strings associated to each goal are subjected to a sequence of preprocessing operations, consisting of the conversion of all characters in lower case and the removal of the unessential elements, namely punctuation, non-ASCII and numerical characters, multiple spacings. The tweet dataset undergoes further preprocessing steps, in which elements that are specifically present in tweets, such as URLs and information on retweets and mentions, are removed. After these operations, the residual text string is partitioned into words and saved in a token list, which is then stripped of stopwords, i.e. terms that do not contain any relevant information on the text content, such as articles, prepositions, conjunctions. Hashtags of the #keyword type, contained in the tweet token lists, are not recognized as part of the NLP libraries dictionary; however, since in many cases hashtags contain the most relevant tweet keywords, it is essential for our analysis to rewrite them in a suitable form, by removing the # symbol and separating the composing lemmas when necessary. For example, the string “#climatechange” is transformed into the two words “climate” and “change”. After correctly codifying hashtags, it is possible to remove from the token list all the terms that do not belong to the dictionary, along with possible strings left empty by the preprocessing operations. Finally, for each item (target or tweet), we obtain a token list, containing all and only the terms with a relevant semantic content. If the token list is empty, the corresponding item is removed from the dataset.

#### Semantic similarities among text items

To measure semantic similarity between two items, we employ *word embedding*, a NLP technique that enables to represent words or phrases as vectors in a multidimensional space, reconstructing their role and context inside a document and identifying relations of semantic and syntactic similarity with other terms (Veremyev et al. 2019). In particular, words that are commonly used in the same contexts correspond to vectors close to each other. The word embedding framework is obtained by training the model on large text corpora through self-supervised machine learning algorithms. One of the most popular and sound implementations of word embedding is represented by the *word2vec* function (Mikolov et al. 2013, 2013) of the *gensim* Python library. This model is based on a neural network trained in a publicly available and very large text database, constituted of around 100 billion words from Google News (Google Open Source Project 2013), which is commonly used as a benchmark in machine learning applications, as it provides an accurate reconstruction of linguistic contexts, interconnections and dynamics characterizing the English language. Given two items (either targets or tweets) $t_1$ and $t_2$ from our SDGs dataset, word embedding allows to represent the respective token lists $l_1$ and $l_2$ as the vectors of real numbers $p^{l_1}=\left( p_{1}^{l_1},\dots ,p_{K}^{l_1}\right)$ and $p^{l_2}=\left( p_{1}^{l_2},\dots ,p_{K}^{l_2}\right)$ in a *K*-dimensional semantic space. The semantic similarity score between $t_1$ and $t_2$ is then computed as the *cosine similarity* between the vectors $p^{l_1}$ and $p^{l_2}$:

1 $$\begin{aligned} sim\left( t_1,t_2\right) = \frac{\sum _{k=1}^{K} p_{k}^{l_1}p_{k}^{l_2}}{\sqrt{\sum _{k=1}^{K}{\left( p_{k}^{l_1}\right) }^2}\sqrt{\sum _{k=1}^{K}{\left( p_{k}^{l_2}\right) }^2}}. \end{aligned}$$

This measure is implemented in the *gensim* library through the *n_similarity* function.

#### Removing spurious tweets through semantic similarity with SDG titles

An additional processing step is necessary in the case of tweets, in order to eliminate erroneously retrieved items, that are compatible with the queries but not related to SDGs. For example, a noise source is represented by tweets containing “SDG*n*”, with *n* a number between 1 and 17, associated to sport events or usernames. These spurious tweets are identified by semantic comparison with the corresponding goal, according to the following procedure. The preprocessing pipeline is applied to the 17 SDG title descriptions [53] to transform them in token lists, that constitute the benchmark to discriminate signal and noise. The comparison between the token lists related to a tweet and the goal title is performed through the *n_similarity* function of the model constructed with the *gensim* library and the *word2Vec* function. We keep in the dataset only those tweets whose semantic similarity with the reference goal is above a given threshold value, which we fix at 0.3, considering the tradeoff between tweet filtering efficiency and preservation of relevant information. The described operations leave 75,070 tweets, namely $84\%$ of the starting value, partitioned among goals as shown in Table 3.

#### Computing inter-goal semantic similarities

The weight of the link between goals $G_i$ and $G_j$, in either the conceptual similarity or the communication similarity network, is obtained in the following way. We first collect the *T*(*i*) token lists $l_{i,1},l_{i,2},\dots ,l_{i,T(i)}$ associated to the goal $G_i$, and the *T*(*j*) lists $l_{j,1},l_{j,2},\dots ,l_{j,T(j)}$ associated to $G_j$. Then, the semantic similarity between each token list related to $G_i$ and each token list related to $G_j$ is computed through the *n_similarity* function. The average of such a set of *T*(*i*)*T*(*j*)/2 similarity measures so obtained is used to weight the link connecting $G_i$ and $G_j$.

### Achievement network of SDGs

The connection between each pair of goals in the achievement network is determined by comparing the related performance networks. In this subsection, we illustrate the procedure to construct performance networks and define the Weighted Jaccard distance used for network comparison.

#### Performance network of countries on a specific goal

To construct the performance networks, we operate a selection of indicators, following criteria of data availability, consistency and non redundant information. The selection is made of the following steps:

- removal of indicators with unavailable values;
- removal of indicators with practically vanishing standard deviation (i.e., $<10^{-5}$);
- removal of binary indicators, that are not suitable for a comparison based on Pearson correlation; binary indicators are 1 for SDG11 and SDG12, 6 for SDG15, and 5 for SDG17;
- computation of Pearson correlation between the available values for each pair of residual indicators; if correlation exceeds 0.98, we retain only the indicator with more available values.

The indicators selected for each goal, whose number is reported in Table 3, are finally normalized in order to compute Pearson correlation between different countries and develop the related performance network. More precisely, to mitigate the effect of outliers, the values exceeding the 99th percentile from above and the 1st percentile from below are replaced by the reference percentiles before performing the linear rescaling of the indicator in [0, 1]. Since the residual indicators are all available for 136 UNMS (see map in the “Appendix”), we choose to include only those countries in the performance networks, in order to have the same node cardinality for all SDGs. Relevant connectivity properties of performance networks are reported in Table 4.

**Table 4:** Features of the performance networks related to each SDG

Goal	Links	Connected components	Nodes in largest component
1	5270	1	136
2	2172	3	134
3	7101	1	136
4	5373	1	136
5	1933	1	136
6	3332	2	135
7	2494	5	132
8	5977	1	136
9	7417	2	135
10	4827	1	136
11	3692	2	135
12	4280	1	136
13	1782	13	124
14	1039	26	109
15	2614	1	136
16	2288	1	136
17	3778	1	136

All networks are characterized by 136 nodes, corresponding to the countries for which all the selected indicators are available

#### Comparing performance networks of countries related to specific goals through weighted Jaccard similarity

We employ Weighted Jaccard (WJAC) similarity (Ioffe 2010; Tantardini et al. 2019) to compare the performance networks of countries, related to different goals. Given two networks $G_{1}$ and $G_{2}$, characterized by the same node set *V*, and edge sets $E_1$ and $E_2$, associated to the adjacency matrices $\{a_{ij}^{1}\}_{i,j\in V}$ and $\{a_{ij}^{2}\}_{i,j\in V}$, respectively, their WJAC similarity is defined as:

2 $$\begin{aligned} J_W\left( A_1,A_2\right) = \left\{ \begin{array}{ccl} &{} \displaystyle \frac{\sum _{i,j \in V} \min \left( a_{ij}^{1},a_{ij}^{2}\right) }{\sum _{i,j \in V} \max \left( a_{ij}^{1},a_{ij}^{2}\right) } &{} \displaystyle {\text {if }} \sum _{i,j \in V} \max \left( a_{ij}^{1},a_{ij}^{2}\right) >0, \\ &{} 1 &{} \displaystyle {\text {if }} \sum _{i,j \in V} \max \left( a_{ij}^{1},a_{ij}^{2}\right) =0. \end{array} \right. \end{aligned}$$

The achievement network is constructed by assigning to each pair of goals a link, whose weight coincides with the WJAC similarity between the two corresponding performance networks.

### Multiplex participation coefficient

The set of three networks considered in our research can be potentially identified as a multiplex of three layers, all with weighted links. However, a direct application of multiplex tools in our framework is dangerous, since it implies the comparison of link weights obtained through non-homogeneous procedures. In order to exploit the multiplex structure, we associate the network on each layer with its binarized counterpart, constructed using the median of the distribution of link weights within the layer as a threshold. Specifically, the binarized version of a given weighted network is obtained by associating an adjacency matrix element 1 to links whose weight is larger than the median, and 0 to other links. Then, to characterize the connectivity distribution of each node *i* among the three layers, we compute the multiplex participation coefficient (Battiston et al. 2014)

3 $$\begin{aligned} P_i = \frac{3}{2} \left[ 1 - \sum _{j=1}^3 \left( \frac{k_i^{[j]}}{k_i^{[1]}+k_i^{[2]}+k_i^{[3]}} \right) ^2 \right] , \end{aligned}$$

where $k_i^{[j]}$ is the node degree in the binarized conceptual $(j=1)$, communication $(j=2)$, and achievement $(j=3)$ networks. The multiplex participation coefficient takes values in [0, 1], being maximal for nodes having an equally relevant degree in all the layers, and minimal for nodes isolated in all but one layer.

**Table 5:** The table reports the weighted degree centrality of nodes corresponding to SDGs in each similarity network along with their multiplex participation coefficient

	Weighted degree centrality			Multiplex participation
Goal	Conceptual	Communication	Achievement	
1	10.080 (11)	8.049 (9)	4.937 (4)	0.927 (10)
2	10.482 (3)	8.042 (10)	3.249 (11)	0.987 (5)
3	9.029 (17)	8.089 (8)	5.090 (1)	0.744 (15)
4	9.854 (12)	7.989 (11)	3.981 (7)	0.947 (9)
5	9.142 (16)	7.642 (16)	2.185 (16)	0.000 (17)
6	9.701 (14)	7.971 (13)	3.748 (9)	0.961 (7)
7	9.685 (15)	8.297 (2)	3.020 (12)	0.883 (11)
8	10.399 (7)	8.334 (1)	5.038 (3)	0.997 (2)
9	11.129 (1)	8.220 (4)	5.082 (2)	0.995 (4)
10	10.093 (10)	8.249 (3)	4.383 (5)	0.977 (6)
11	10.307 (8)	7.975 (12)	2.714 (14)	0.830 (13)
12	10.462 (5)	8.138 (6)	4.330 (6)	0.996 (3)
13	10.508 (2)	8.090 (7)	2.416 (15)	0.851 (12)
14	10.307 (9)	7.743 (15)	1.658 (17)	0.639 (16)
15	10.474 (4)	7.922 (14)	3.850 (8)	0.954 (8)
16	9.702 (13)	7.634 (17)	2.901 (13)	0.797 (14)
17	10.441 (6)	8.175 (5)	3.702 (10)	0.997 (1)

The weighted degree centrality coincides with the sum of link weights connected to each node, while the multiplex participation coefficient is computed from the binarized versions of the similarity networks on each layer, as described in Eq. (3)

## Figures and Tables

**Fig. 5:**
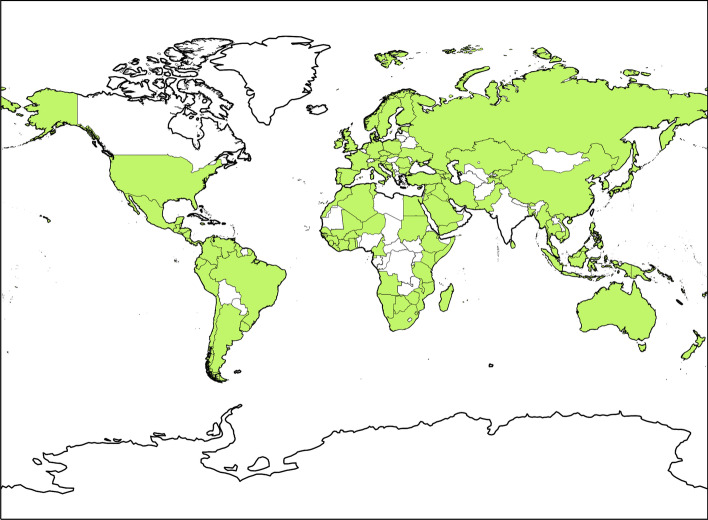
Map illustrating countries included as nodes in performance networks, highlighted in green

**Fig. 6:**
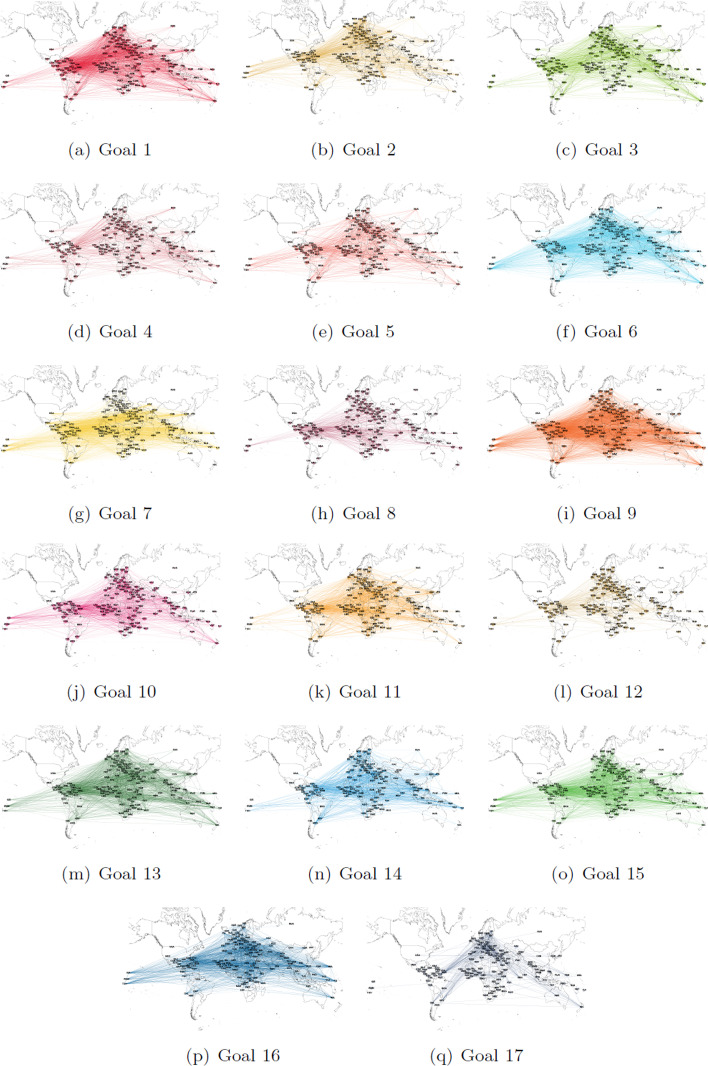
Graphical representation of the SDG performance networks, related to each SDG, in which nodes correspond to UNMS. These networks, compared with each other through the WJAC similarity, provide the basis to construct the SDG achievement network. Each network reports only the links whose weight is larger that 0.8

## Data Availability

The data that support the findings of this study are either publicly available on databases cited in the bibliography, or available from the corresponding author upon reasonable request.

## Appendix

In Table 5, we report the values of weighted degree centrality of nodes in each of the three examined SDG networks, along with the multiplex participation coefficient of each node.

Figure 5 shows the geographical map illustrating the 136 UNMS included as nodes in the 17 country performance networks (one for each goal). These networks, represented in Fig. 6, are compared with each other through the WJAC similarity, and provide the basis to construct the SDG achievement network, as described in the Materials and Methods section.

## Disclaimer

The designations employed and the presentation of the material in this paper do not imply the expression of any opinion whatsoever on the part of the United Nations concerning the legal status of any country, territory, city or area, or of its authorities, or concerning the delimitation of its frontiers or boundaries. The designations “developed” and “developing” economics are intended for statistical convenience and do not necessarily imply a judgment about the state reached by a particular country or area in the development process. The term “country” as used in the text of this publication also refers, as appropriate, to territories or areas. The views expressed are those of the individual authors of the paper and do not imply any expression of opinion on the part of the United Nations.

## Abbreviations

SDG: Sustainable Development Goal
UN: United Nations
UNMS: United Nations Member State(s)
NLP: Natural Language Processing
WJAC: Weighted Jaccard
